# Effects of Exercise-Induced Neuromuscular Fatigue on Jump Performance and Lower-Limb Asymmetries in Youth Female Team Sport Athletes

**DOI:** 10.5114/jhk/174073

**Published:** 2023-10-27

**Authors:** Azahara Fort-Vanmeerhaeghe, Chris Bishop, Alicia M. Montalvo, Bernat Buscà, Jordi Arboix-Alió

**Affiliations:** 1 Faculty of Psychology, Education Sciences and Sport (FPCEE) Blanquerna, Ramon Llull University, Barcelona, Spain.; 2 School of Health Sciences (FCS) Blanquerna, Ramon Llull University, Barcelona, Spain.; 3 Segle XXI Female Basketball Team, Catalan Federation of Basketball, Esplugues de Llobregat, Spain.; 4 Faculty of Science and Technology, London Sport Institute, Middlesex University, London, UK.; 5 College of Health Solutions, Arizona State University, Phoenix, AZ, USA.

**Keywords:** symmetry, fatigue, women, adolescents

## Abstract

The objective of the present study was to examine the effect of acute neuromuscular fatigue on unilateral jump performance and inter-limb asymmetries. Thirty elite youth female team sport athletes (age: U-14 to U-18) performed the Unilateral Countermovement Jump (UCJ) and the Unilateral Drop Jump (UDJ) (18-cm box) tests before and approximately 10 minutes after the 30-15 intermittent fitness test (30-15 IFT). A paired samples t-test showed significant reductions in UCJ jump height in the right leg after the 30-15 IFT (p = 0.018; d = 0.33), but not in the left leg (p = 0.459; d = 0.48). For the UDJ, significant reductions in jump height were shown in both the right (p < 0.001; d = 0.33) and left (p < 0.001; d = 0.33) legs. In addition, for the reactive strength index (UDJ), significant reductions were seen in the left leg after the 30-15 IFT (p < 0.001; d = 0.31), but not in the right leg (p = 0.948; d < 0.001). Only UCJ inter-limb jump height asymmetries increased significantly post 30-15 IFT (p = 0.033; d = 0.46). In conclusion, the current study indicates that the 30-15 IFT provides a sufficient dose of activity for inducing acute fatigue in elite youth female team sport athletes. Therefore, monitoring jump height in unilateral jump testing is recommended given the tests’ sensitivity to detect significant differences in physical performance and inter-limb asymmetries under acutely fatigued conditions in healthy youth female athletes.

## Introduction

In recent years, participation in women’s team sports has increased in popularity, and has been accompanied by increases in external loads during competition. However, such increases are likely to lead to a greater risk of lower limb non-contact injuries during training and competition if athletes are not sufficiently physically prepared (Collings et al., 2021). In female athletes specifically, the risk of injury is greater than that of males for conditions such as anterior knee pain (Fagan and Delahunt, 2008), ACL rupture (Paterno et al., 2010) and ankle sprains (Hosea et al., 2000). Despite the complexity of sport injury etiology, this increased risk in female athletes has been related to neuromuscular risk factors such as fatigue (Heil et al., 2020) and the predominance of one leg over the other in terms of strength and power (i.e., inter-limb asymmetries) (Maloney, 2019).

Within the context of sport, fatigue can be defined as a process in which there is a progressive decrease in objectively measured physical output (Enoka and Duchateau, 2008). In addition, fatigue is associated with changes in neuromuscular control strategies during sport-specific tasks, such as decreases in strength (Greig and Siegler, 2009), decreased dynamic joint stability (Chappell et al., 2005), reduced proprioception (Forestier et al., 2002), and negative changes to biomechanics of the lower extremities (Borotikar et al., 2008). Team sport athletes must adapt to conditions of fatigue to cope with high training and competition volumes that result from sport seasons. In fact, fatigue has been shown to both decrease match performance (Bradley et al., 2013) and increase the risk of non-contact injuries, such as ACL injuries (Brazen et al., 2010) and ankle sprains (Forestier et al., 2002). Moreover, it is important to highlight that fatigue seems to increase the risk of injury in female athletes more compared to male athletes (Kernozek et al., 2008). Thus, quantifying fatigue and understanding how our key performance indictors respond represent an important piece of the athlete monitoring puzzle for practitioners.

Team sports are characterized by repetitive and intermittent unilateral explosive actions, such as jumping, sprinting, throwing, etc., which often occur during crucial moments in competition (Spencer et al., 2005). Many of these high-intensity unilateral actions could lead to the development of asymmetries in neuromuscular adaptations of the lower limbs (Bishop et al., 2021a), especially seeing as it is unlikely that each limb will experience the same volume of high-intensity actions. Similarly to fatigue, inter-limb asymmetries have received special attention in recent years as they have been associated with both a decrease in physical performance (Bishop et al., 2018a) and an increase in the risk of non-contact injuries (Fort-Vanmeerhaeghe et al., 2022; Paterno et al., 2010). However, there is a lack of literature on how fatigue affects inter-limb asymmetries in team sport athletes, particularly in female athletes (Bishop et al., 2022). Bishop et al. (2022) observed how the group mean asymmetry value (from a unilateral countermovement and a drop jump) rarely changed immediately post-match in elite youth soccer players. However, real changes in asymmetry were evident at the individual level, when compared to the baseline coefficient of variation (CV). Furthermore, the direction of asymmetry showed little consistency (Kappa: −0.20 to 0.60), when comparing limb dominance scores between baseline and post-match. In contrast, Bromley et al. (2018) showed how jump performance was reduced and between-limb differences significantly increased when comparing pre- to post-match performance in adolescent male soccer players. Moreover, the same group of authors showed how multiple repeated sprints (6 x 40 m with 20 s of rest between each sprint) reduced single leg countermovement jump height and increased inter-limb jump asymmetries after each set of repeated sprints in recreationally active adult males (Bishop et al., 2021b), although statistical significance was only reached for asymmetry after the third set of the protocol. However, to the best of our knowledge, only one study in adult female soccer players has been conducted on the topic of effects of fatigue on performance. The results of that study showed no significant differences in inter-limb asymmetries after a field test simulating soccer-specific movement patterns (Delextrat et al., 2013).

Neuromuscular fatigue has traditionally been examined with laboratory-based tasks during isometric, concentric or eccentric actions (Nicol et al., 2006). However, these protocols are not sport-specific and do not mimic what team sport athletes are required to do in game scenarios, where most actions are characterized by jumping, hopping or changing direction (Spencer et al., 2005). In these types of actions, muscle function is characterized by the stretch-shortening cycle (SSC), which consists of a fast action of muscle stretching (eccentric action) followed by a rapid shortening phase (concentric action) (Verkhoshansky, 2009). Thus, to optimize ecological validity for test measures in athletes from these sports, neuromuscular fatigue (and subsequent asymmetry outcomes) should be explored during SSC tasks, such as jumping. Understanding the potential effects of fatigue on the neuromuscular function of the lower limbs will enable sport performance practitioners to better plan and optimize training loads. Therefore, the aim of this study was to examine the effect of acute neuromuscular fatigue on unilateral jump performance and inter-limb asymmetries. It was hypothesized that acute fatigue would result in decreased jump performance and increased inter-limb asymmetries.

## Methods

The present study used a prospective, experimental pretest-posttest design to compare jump performance and inter-limb asymmetries before and after an intermittent test (30-15 intermittent fitness test, 30-15 IFT) in a group of elite female basketball and handball players. The Unilateral Countermovement Jump (UCJ) and the Unilateral Drop Jump (UDJ) tests were performed before and approximately ten minutes after the 30-15 IFT. All tests were performed in May 2021, at the end of the sport season.

### Participants

Thirty elite youth female team-sport athletes from basketball (n = 22), and handball (n = 8), took part in this study (Table 1). All athletes were actively participating in a four-year national professional development program training and studying in the same high-performance sports center, in Esplugues de Llobregat (Barcelona). Written parental and participant’s consent was acquired before the study started. This study was certified by the Ramon Llull University Ethics Committee (approval number: 1718007D) and conformed to the recommendations of the Declaration of Helsinki.

**Table 1:** Participants’ characteristics by sport.

Sport	n	Age (years)	Height (m)	Mass (kg)	BMI (kg•m^2^)	Training experience (years)	Years post PHV *
Basketball	22	16.41 ± 1.13	1.80 ± 0.07	69.27 ± 9.15	21.28 ± 2.07	8.09 ± 1.93	4.54 ± 0.79
Handball	8	15.9 ± 0.97	1.70 ± 0.05	63.25 ± 6.76	21.77 ± 1.83	7.50 ± 1.31	3.99 ± 0.5
Total	30	16.29 ± 1.11	1.78 ± 0.08	67.66 ± 8.89	21.41 ± 1.99	7.93 ± 1.78	4.39 ± 0.75

* Peak Height Velocity (PHV), estimation of biological age (Mirwald et al., 2002)

### Design and Procedures

Athletes attended two sessions over 14 days; the first was a familiarization session, and the second one was for data collection. They performed the same neuromuscular-type warm-up before all measurements, which included mobility exercises, displacements, and jumps, which gradually progressed in intensity. After the warm-up, athletes completed two maximal effort attempts for each test. All tests were performed at random with one-minute of rest between trials. Athletes completed three trials on each limb and the greatest score was recorded for data analysis. The warm-up procedures were not repeated after the 30-15 IFT protocol.

### Unilateral Countermovement Jump

The UCJ test was registered on a contact mat (Chronojump Boscosystem, Barcelona, Spain) using Chronojump software to estimate the vertical jump height (Bosco et al., 1983). Players stood on one limb with hands on hips, descended to a self-selected depth, and then rapidly extended the stance limb to jump vertically. Participants were informed to “jump as high as possible” for every test.

### Unilateral Drop Jump

The UDJ was also performed using a contact mat (Chronojump Boscosystem, Barcelona, Spain). Athletes started on an 18-cm box. With hands on hips, they hopped off the box with the chosen limb and rebounded off the ground using the same limb. They were instructed to minimize ground contact time and maximize jump height (Bishop et al., 2022). Jump height, ground contact time (GCT), and the reactive strength index (RSI) (quantified as flight time divided by GCT) were recorded.

### 30-15 Intermittent Fitness Test

The 30-15 IFT is a valid and reliable test created as a reference guide for interval training prescription and to measure typical physical capacities that are challenged during intermittent sports, such as handball, basketball, and soccer (Buchheit, 2008). In the study, the modified version of the 30-15 intermittent fitness test (Haydar et al., 2011) was performed on a 28-m long basketball court. The 30-15 IFT combined 30-s shuttle runs interspersed with 15-s walking recovery time. The test started at 8 km/h and speed was augmented 0.5 km/h every stage. The fastest speed (latest stage) reached by each athlete was used for subsequent data analysis.

### Statistical Analysis

Data are presented as mean and standard deviation (SD). Data were assessed for normality using the Shapiro-Wilk test. Within-session reliability of recorded data was analyzed using a two-way random intraclass correlation coefficient (ICC) with an absolute agreement (95% confidence intervals) and the coefficient of variation (CV). ICC values were interpreted according to Koo and Li (2006). CV values were to be satisfactory if less than 10% (Cormack et al., 2008). Paired sample *t*-tests were used to determine whether unilateral jump test heights (normally distributed) were significantly different between pre- and post-30-15 IFT, with statistical significance established at *p* < 0.05. However, asymmetry scores were not normally distributed, thus Wilcoxon Signed-Rank tests were used to identify significant differences between pre- and post-30-15 IFT. The magnitude of differences between pre- and post-tests was also computed via Cohen’s *d* effect sizes (ESs). To determine the magnitude of asymmetry, the asymmetry index (ASI) was calculated as described by Impellizzeri et al. (2007) and Fort-Vanmeerhaeghe et al. (2015).

To determine the direction of asymmetry, an “IF function” was included at the end of the formula in Microsoft Excel: *IF(left <right,1,−1) (Bishop et al., 2018b). In doing so, athletes that had a change in asymmetry greater than the pre-30-15 IFT CV were also recognized as presenting a real change (Bishop et al., 2022).

The highest performing limb was determined as the leg with the greatest value in each jump. To determine the levels of agreement for how consistently an asymmetry favored the same side (direction of asymmetry, right or left), the Kappa coefficient (κ) was calculated to compare pre- and post-UCJ and UDJ inter-limb asymmetries. Kappa values were interpreted according to Viera and Garret (2005).

## Results

Table 2 shows descriptive statistics and reliability measures for UCJ and UDJ tests. Nearly all the tests showed excellent within-session ICC values (≥ 0.9) and had acceptable consistency with CV values < 10%.

**Table 2:** Mean test scores ± SD, reliability measures, and comparison of pre- and post-30-15 IFT for the SLCMJ and SLDJ (paired samples *t*-tests).

			Mean (SD)	ICC (95% CI)	CV (%)	*p*	Effect size *(d*)
SLCMJ (m)	*Right*	*PRE*	0.16 (0.03)	0.90 (0.83–0.95)	4.87	0.018*	0.33
		*POST*	0.15 (0.03)	0.90 (0.83–0.95)	4.93		
	*Left*	*PRE*	0.16 (0.03)	0.91 (0.85–0.95)	4.45	0.459	0.48
		*POST*	0.18 (0.05)	0.93 (0.86–0.95)	7.62		
SLDJ (m)	*Right*	*PRE*	0.14 (0.03)	0.85 (0.79–0.94)	8.26	0.000*	0.33
		*POST*	0.13 (0.03)	0.90 (0.83–0.95)	6.48		
	*Left*	*PRE*	0.14 (0.03)	0.85 (0.74–0.92)	5.73	0.000*	0.33
		*POST*	0.13 (0.03)	0.86 (0.76–0.93)	9.18		
SLDJ RSI	*Right*	*PRE*	1.14 (0.26)	0.87 (0.78–0.93)	5.92	0.948	0.00
		*POST*	1.14 (0.25)	0.88 (0.79–0.93)	6.23		
	*Left*	*PRE*	1.15 (0.22)	0.86 (0.76–0.93)	5.16	0.000*	0.31
		*POST*	1.08 (0.22)	0.89 (0.81–0.94)	4.90		

SLCMJ = Single Leg Countermovement Jump; SLDJ = Single Leg Drop Jump; RSI = Reactive Strength index; SD = standard deviation; ICC = intraclass correlation coefficient; CI = confidence intervals; CV = coefficient of variation; *d* = Cohen's d, * p < 0.05.

**Figure 1:**
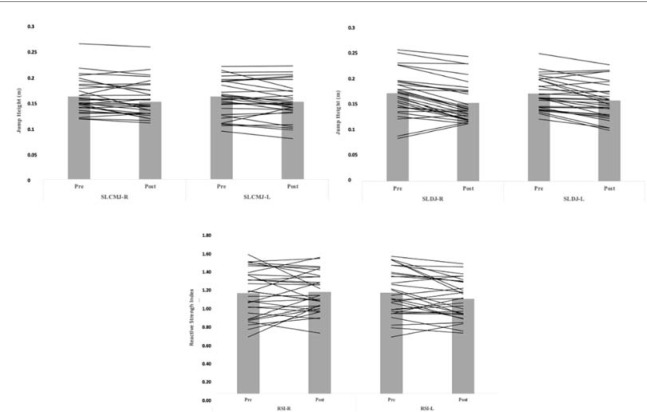
Individual performance data during pre and post 30-15 IFT for the single-leg countermovement jump height (top left), single-leg drop jump height (top right) and the reactive strength index (bottom).

For the UCJ, significant reductions in jump height were observed in the right leg after the 30-15 IFT (*p* = 0.018; *d* = 0.33), but not in the left leg (*p* = 0.459; *d* = 0.48). For the UDJ, significant reductions in jump height were observed in the right (*p* = 0.000; *d* = 0.33) and left (*p* = 0.000; *d* = 0.33) legs. In addition, for the RSI (UDJ), significant reductions were seen in the left leg after the 30-15 IFT (*p* = 0.000; *d* = 0.31), but not in right leg (*p* = 0.948; *d* = 0.001). Figure 1 shows individual data pre- and post-30-15 IFT for the UCJ, the UDJ and the RSI.

Table 3 shows pre- vs. post-30-15 IFT inter-limb ASI values. For the UCJ, the ASI increased significantly from pre- to post-30-15 IFT (*p* = 0.033; *d* = 0.46). No differences were observed between pre- and post-30-15 IFT for the UDJ (*p* = 0.484; *d* = 0.18) and the RSI (*p* = 0.393; *d* = 0.03).

**Table 3:** SLCMJ and SLDJ inter-limb asymmetries before and after the 30-15 IFT.

ASI (%)		Mean (SD)	*p*	Effect size *(d*)
*SLCMJ*	*PRE*	9.37 (6.08)	0.033*	0.46
	*POST*	14.86 (15.36)		
*SLDJ*	*PRE*	11.34 (10.17)	0.484	0.18
	*POST*	9.81(6.66)		
*RSI*	*PRE*	10.39 (8.89)	0.393	0.03
	*POST*	10.17 (7.72)		

ASI (%) = Asymmetry index; SLCMJ = Single Leg Countermovement Jump; SLDJ = Single Leg Drop Jump; RSI = Reactive Strength index; SD = standard deviation; *d* = Cohen’s d, * p < 0.05.

Consistency in limb preference from pre- to post-30-15 IFT for each test was moderate or lower. For the UCJ, limb agreement was moderate (kappa: 0.53). For the UDJ, limb agreement was fair (kappa: 0.39). For the RSI, limb agreement was moderate (kappa: 0.53).

**Figure 2:**
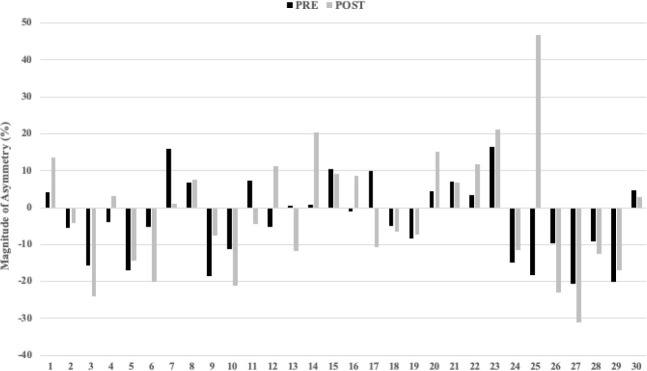
Individual inter-limb asymmetry data during the pre and post 30-15 IFT for the single-leg countermovement jump test height.

**Figure 3:**
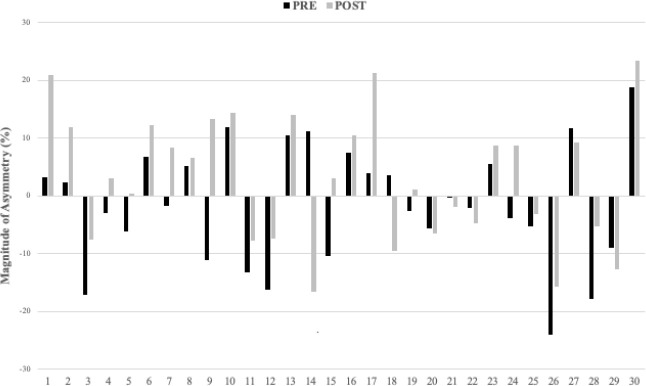
Individual inter-limb asymmetry data during the pre and post 30-15 IFT for the single-leg drop jump test height.

**Figure 4:**
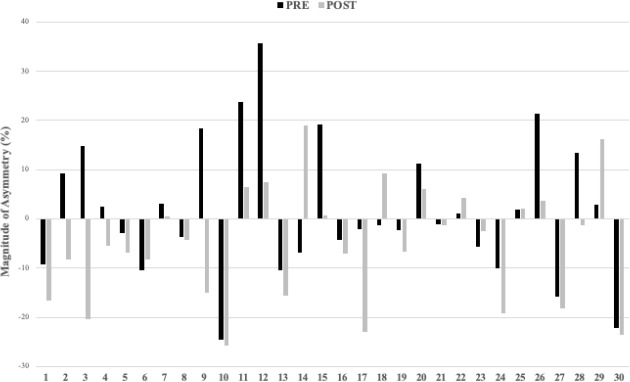
Individual inter-limb asymmetry data during the pre and post 30-15 IFT for the reactive strength index.

Figures 2–4 show the highly individual and variable nature of inter-limb asymmetry data from pre- to post-30-15 IFT for the UCJ, the UDJ and the RSI. In addition, changes in asymmetry (between pre- and post-30-15 IFT) greater than the pre-test CV values varied between tests. Of the 30 athletes tested, real changes in the ASI ranged from 18 for UCJ height, 17 for UDJ height, to 15 for the RSI.

## Discussion

The aim of this study was to examine the effect of acute neuromuscular fatigue on unilateral jump performance and inter-limb asymmetries. Results showed significant reductions in performance after the 30-15 IFT for UCJ height in the right leg, UDJ height for both legs, and the RSI in the left leg. These findings demonstrate the high sensitivity of the UDJ for detecting fatigue-related changes in performance. Regarding inter-limb jump height asymmetries (magnitude), only UCJ height increased significantly post-30-15 IFT compared to pre-30-15 IFT. This finding demonstrates the high sensitivity of the UCJ for detecting fatigue-related changes in the magnitude of asymmetry in youth athletes. Moreover, it is also important to look at the directionality, where 7 out of 30 participants showed changes in the direction of asymmetry, which represents 23% of the sample (Figure 2). In this case, consistency in the direction of asymmetry occurred more often than changes. However, it was highly individualized.

### Effects of Acute Fatigue on Unilateral Jump Performance

The findings of this study show that unilateral jump performance, specifically UCJ height in the right leg (ES = 0.33), UDJ height in both legs (ES = 0.33 in the right leg; ES = 0.33 in the left leg) and the RSI in the left leg (ES = 0.31), is negatively impacted by acute fatigue. Similarly to our results, Bishop et al. (2022) demonstrated changes in UCJ jump height in both limbs and in sprint speed following sets of repeated sprints in recreationally active males. Moreover, the same group of authors showed UCJ and UDJ heights decreased after matches in adolescent male soccer players (Bishop et al., 2022). Although the sample of the aforementioned study differed from our participants, the methods and protocols for measuring unilateral jumping height were very similar. Thus, it is possible that jump measures (i.e., jump height) are more sensitive to true change when performed unilaterally compared to bilaterally, which has been noted in previous unilateral jump research.

Despite these results, caution should be used in the interpretation and application of the findings. Figure 1 shows the high variability in changes when comparing jump performance before and after the fatigue protocol. Differences were not consistent across athletes, indicating that fatigue is an individual, multi-faceted process. Thus, practitioners who implement these tests should interpret data on an individual basis as it may not be useful to aggregate findings.

### Effects of Acute Fatigue on the ASI

Despite the individual and variable nature of asymmetries (Figures 2–4), inter-limb asymmetry measures increased significantly for UCJ height (*p* = 0.033; *d* = 0.46) when comparing pre- (9.37 ± 6.08) and post-fatigue intervention (14.36 ± 15.36) values. Otherwise, no significant differences were observed in inter-limb asymmetries from pre- to post-fatigue intervention for UDJ height and the RSI. In accordance with our results, some studies showed how the UCJ inter-limb asymmetries increased significantly after a soccer competition in elite adolescent male athletes (Bromley et al., 2018) and after repeated sprints in recreationally active male athletes (Bishop et al., 2021b). Contrary to our results, Delextrat et al. (2013) showed no significant differences in inter-limb asymmetries following a field test simulating soccer-specific movement patterns in female soccer athletes. The differences in results could be explained by their use of isokinetic strength assessments of the hamstrings and quadriceps to calculate asymmetries between limbs. Isokinetic measurements are not plyometric (i.e., no stretch-shortening cycle) and the movements are less specific to the movement patterns seen in most team sports. When interpreting asymmetries, it is important to consider the instruments and outcomes used, and the skill-specific nature of asymmetries (Arboix-Alió et al., 2021; Fort-Vanmeerhaeghe et al., 2015). To our knowledge, our study is the first one to examine the effects of an acute fatigue protocol on interlimb jump asymmetries in elite youth female team sport athletes. One possible explanation for the increased sensitivity of the UDJ relative to the UCJ is complexity. The UDJ is a more complex and specific task than the UCJ because the SSC occurs faster. This difference in speed translates to fewer opportunities for an athlete to manipulate their jump strategy, and may be the reason for differences in ASI values when comparing the two tests (Bishop et al., 2022). Although players were familiar with both tests, the UDJ is a challenging task even for elite athletes, which could make generalizing the data less reliable. This idea is further supported by the higher CV values that accompanied the UDJ relative to the UCJ (Table 2). Consequently, practitioners need to ensure they have sufficiently familiarized athletes with the UDJ to obtain reliable data.

### Real Changes to the RSI and UDJ Height

Recent research has demonstrated the importance of reporting inter-limb asymmetries in conjunction with measures of variability (i.e., CV), as this enables practitioners to determine what may be considered a “real change” in their data (Bishop et al., 2022; Exell et al., 2017) beyond group mean values. In this study, the range in real change in asymmetry over baseline CV values varied from 60% of the sample for UCJ height, 56.67 % for UDJ height, and 50% for the RSI. Thus, despite the fact that there were no significant changes in the UDJ or RSI asymmetries from pretest to posttest, it is clear that over half of the sample exhibited true changes in asymmetry. This finding further highlights the need to assess side-to-side differences on an individual basis (Bishop et al., 2021a). Finally, when considering our primary aim of determining the effects of a fatiguing protocol, monitoring such changes on an individual basis may help identify ‘responders and non-responders’ to particular training protocols. In turn, this may help practitioners make decisions about whether athletes should scale back on training if persistent reductions in performance are evident.

### Inconsistencies in the Highest Performing Limb

There were inconsistencies in the highest performing limb from pre- to post-fatigue in all tests. For the UCJ, levels of agreement were moderate (kappa: 0.53); for the UDJ height fair (kappa: 0.39); and for the RSI moderate (kappa: 0.53). These differences in directionality highlight the variable nature of inter-limb asymmetries supported by the scientific literature (Bishop et al., 2018a).

### Limitations

Although this study offers useful findings, some limitations should be acknowledged. First, we currently know that to improve performance and reduce the risk of injury from high-load joint actions, such as jumps and changes of direction, it is not sufficient to only analyze jump height or the RSI. It is also important to assess the kinetics and kinematics of the lower extremity during high-intensity actions to evaluate the different neuromuscular control strategies applied by athletes under fatigue conditions. Second, the current findings may not be generalizable to other populations. The nature of inter-limb asymmetries is individual; thus, our findings may only apply to elite youth female team sports athletes. Third, despite the fact that the 30-15 IFT appeared to be effective at inducing acute fatigue, it does not fully mimic the reality of a practice or competition. In fact, fatigue in team sport athletes is a complex process that develops progressively and not as a point of acute muscular failure (Marqués-Jiménez et al., 2017). Thus, future research should focus on implementing these testing procedures after real practice or competition.

## Conclusions

Though not an aim, the results of this study showed that the 30-15 IFT was an effective fatigue protocol to reduce jump performance and increase inter-limb asymmetries in a group of youth female team sport athletes. Additionally, unilateral jump testing for height and limb asymmetries can be used to monitor fatigue and performance changes in elite youth female team sport athletes, though the problem of inconsistency of highest performing limb persists. However, given the variable and individual nature of inter-limb asymmetries (high SD values), practitioners can consider utilizing unilateral jumps as they are sensitive instruments for detecting fatigue. To reduce the risk of injury and performance decrements from fatigue practitioners should train sport actions under conditions of fatigue to mimic the reality of competition.

## References

[ref1] Arboix-Alió, J., Bishop, C., Benet, A., Buscà, B., Aguilera-Castells, J., & Fort-Vanmeerhaeghe, A. (2021). Assessing the Magnitude and Direction of Asymmetry in Unilateral Jump and Change of Direction Speed Tasks in Youth Female Team-Sport Athletes. Journal of Human Kinetics, 79(1), 15–27. 10.2478/hukin-2021-0061 34400983 PMC8336540

[ref2] Bishop, C., Lake, J., Loturco, I., Papadopoulos, K., Turner, A., & Read, P. (2021a). Interlimb Asymmetries: The Need for an Individual Approach to Data Analysis. Journal of Strength and Conditioning Research, 35(3), 695–701. 10.1519/JSC.0000000000002729 33587548

[ref3] Bishop, C., McAuley, W., Read, P., Gonzalo-Skok, O., Lake, J., & Turner, A. (2021b). Acute Effect of Repeated Sprints on Interlimb Asymmetries During Unilateral Jumping. Journal of Strength and Conditioning Research, 35(8), 2127–2132. 10.1519/JSC.0000000000003109 30865058

[ref4] Bishop, C., Read, P., Lake, J., Chavda, S., & Turner, A. (2018a). Interlimb asymmetries: Understanding how to calculate differences from bilateral and unilateral tests. Strength and Conditioning Journal, 40(4), 1–6. 10.1519/SSC.0000000000000371

[ref5] Bishop, C., Read, P., Stern, D., & Turner, A. (2022). Effects of Soccer Match-Play on Unilateral Jumping and Interlimb Asymmetry: A Repeated-Measures Design. Journal of Strength and Conditioning Research, 36(1), 193–200. 10.1519/JSC.0000000000003389 31985557

[ref6] Bishop, C., Turner, A., & Read, P. (2018b). Training methods and considerations for practitioners to reduce interlimb asymmetries. Strength and Conditioning Journal, 40(2), 40–46. 10.1519/SSC.0000000000000354

[ref7] Borotikar, B. S., Newcomer, R., Koppes, R., & McLean, S. G. (2008). Combined effects of fatigue and decision making on female lower limb landing postures: central and peripheral contributions to ACL injury risk. Clinical Biomechanics, 23(1), 81–92. 10.1016/j.clinbiomech.2007.08.008 17889972

[ref8] Bosco, C., Luhtanen, P., & Komi, P. V. (1983). A simple method for measurement of mechanical power in jumping. European Journal of Applied Physiology and Occupational Physiology, 50(2), 273–282. 6681758 10.1007/BF00422166

[ref9] Bradley, P. S., Carling, C., Gomez Diaz, A., Hood, P., Barnes, C., Ade, J., Boddy, M., Krustrup, P., & Mohr, M. (2013). Match performance and physical capacity of players in the top three competitive standards of English professional soccer. Human Movement Science, 32(4), 808–821. 10.1016/j.humov.2013.06.002 23978417

[ref10] Brazen, D. M., Todd, M. K., Ambegaonkar, J. P., Wunderlich, R., & Peterson, C. (2010). The effect of fatigue on landing biomechanics in single-leg drop landings. Clinical Journal of Sport Medicine, 20(4), 286–292. 20606514 10.1097/JSM.0b013e3181e8f7dc

[ref11] Bromley, T., Turner, A., Read, P., Lake, J., Maloney, S., Chavda, S., & Bishop, C. (2021). Effects of a Competitive Soccer Match on Jump Performance and Interlimb Asymmetries in Elite Academy Soccer Players. Journal of Strength and Conditioning Research, 35(6), 1707–1714. 10.1519/JSC.0000000000002951 34027923

[ref12] Buchheit, M. (2008). The 30-15 Intermittent Fitness Test: Accuracy for Individualizing Interval Training of Young Intermittent Sport Players. Journal of Strength and Conditioning Research, 22(2), 365–374. 10.1519/JSC.0b013e3181635b2e 18550949

[ref13] Chappell, J. D., Herman, D. C., Knight, B. S., Kirkendall, D. T., Garrett, W. E., & Yu, B. (2005). Effect of fatigue on knee kinetics and kinematics in stop-jump tasks. American Journal of Sports Medicine, 33(7), 1022–1029. 15983125 10.1177/0363546504273047

[ref14] Collings, T. J., Bourne, M. N., Barrett, R. S., du Moulin, W., Hickey, J. T., & Diamond, L. E. (2021). Risk Factors for Lower Limb Injury in Female Team Field and Court Sports: A Systematic Review, Meta-analysis, and Best Evidence Synthesis. Sports Medicine, 51(4), 759–776. 10.1007/s40279-020-01410-9 33400215

[ref15] Cormack, S. J., Newton, R. U., McGulgan, M. R., & Doyle, T. L. A. (2008). Reliability of measures obtained during single and repeated countermovement jumps. International Journal of Sports Physiology and Performance, 3, 131–134. 10.1123/ijspp.3.2.131 19208922

[ref16] Delextrat, A., Baker, J., Cohen, D. D., & Clarke, N. D. (2013). Effect of a simulated soccer match on the functional hamstrings-to-quadriceps ratio in amateur female players. Scandinavian Journal of Medicine and Science in Sports, 23(4), 478–486. 10.1111/j.1600-0838.2011.01415.x 22107131

[ref17] Enoka, R. M., & Duchateau, J. (2008). Muscle fatigue: what, why and how it influences muscle function. Journal of Physiology, 586(1), 11–23. 17702815 10.1113/jphysiol.2007.139477 PMC2375565

[ref18] Exell, T., Irwin, G., Gittoes, M., & Kerwin, D. (2017). Strength and performance asymmetry during maximal velocity sprint running. Scandinavian Journal of Medicine and Science in Sports, 27(11), 1273–1282. 10.1111/sms.12759 27671707

[ref19] Fagan, V., & Delahunt, E. (2008). Patellofemoral pain syndrome: a review on the associated neuromuscular deficits and current treatment options. British Journal of Sports Medicine, 42(10), 789–795. 18424487 10.1136/bjsm.2008.046623

[ref20] Forestier, N., Teasdale, N., & Nougier, V. (2002). Alteration of the position sense at the ankle induced by muscular fatigue in humans. Medicine and Science in Sports and Exercise, 34(1), 117–122. 11782656 10.1097/00005768-200201000-00018

[ref21] Fort-Vanmeerhaeghe, A., Milà-Villarroel, R., Pujol-Marzo, M., Arboix-Alió, J., & Bishop, C. (2022). Higher Vertical Jumping Asymmetries and Lower Physical Performance are Indicators of Increased Injury Incidence in Youth Team-Sport Athletes. Journal of Strength and Conditioning Research, 36(8), 2204–2211. 10.1519/JSC.0000000000003828 33009354

[ref22] Fort-Vanmeerhaeghe, A., Montalvo, A. M., Sitjà-Rabert, M., Kiefer, A. W., & Myer, G. D. (2015). Neuromuscular asymmetries in the lower limbs of elite female youth basketball players and the application of the skillful limb model of comparison. Physical Therapy in Sport, 16(4), 317–323. 26093377 10.1016/j.ptsp.2015.01.003

[ref23] Greig, M., & Siegler, J. C. (2009). Soccer-Specific Fatigue and Eccentric Hamstrings Muscle Strength. Journal of Athletic Training, 44(2), 180–184. 10.4085/1062-6050-44.2.180 19295963 PMC2657020

[ref24] Haydar, B., al Haddad, H., Ahmaidi, S., & Buchheit, M. (2011). Assessing inter-effort recovery and change of direction ability with the 30-15 intermittent fitness test. Journal of Sports Science and Medicine, 10(2), 346–354. 24149882 PMC3761847

[ref25] Heil, J., Loffing, F., & Büsch, D. (2020). The Influence of Exercise-Induced Fatigue on Inter-Limb Asymmetries: a Systematic Review. Sports Medicine-Open, 6(1), 39. 10.1186/s40798-020-00270-x 32844254 PMC7447715

[ref26] Hosea, T. M., Carey, C. C., & Harrer, M. F. (2000). The gender issue: epidemiology of ankle injuries in athletes who participate in basketball. Clinical Orthopaedics and Related Research, 372, 45–49. 10.1097/00003086-200003000-00006 10738413

[ref27] Impellizzeri, F. M., Rampinini, E., Maffiuletti, N., & Marcora, S. M. (2007). A vertical jump force test for assessing bilateral strength asymmetry in athletes. Medicine and Science in Sports and Exercise, 39(11), 2044–2050. 10.1249/mss.0b013e31814fb55c 17986914

[ref28] Kernozek, T. W., Torry, M. R., & Iwasaki, M. (2008). Gender differences in lower extremity landing mechanics caused by neuromuscular fatigue. The American Journal of Sports Medicine, 36(3), 554–565. 18006677 10.1177/0363546507308934

[ref29] Koo, T. K., & Li, M. Y. (2016). A Guideline of Selecting and Reporting Intraclass Correlation Coefficients for Reliability Research. Journal of Chiropractic Medicine, 15(2), 155–163. 10.1016/j.jcm.2016.02.012 27330520 PMC4913118

[ref30] Maloney, S. J. (2019). The Relationship Between Asymmetry and Athletic Performance. Journal of Strength and Conditioning Research, 33(9), 2579–2593. 10.1519/JSC.0000000000002608 29742749

[ref31] Marqués-Jiménez, D., Calleja-González, J., Arratibel, I., & Delextrat, A. (2017). Fatigue and Recovery in Soccer : Evidence and Challenges. Open Sports Journal Science, 10, 52–70. 10.2174/1875399X01710010051

[ref32] Nicol, C., Avela, J., & Komi, P. V. (2006). The Stretch-Shortening Cycle. Sports Medicine, 36(11), 977–999. 10.2165/00007256-200636110-00004 17052133

[ref33] Paterno, M. V, Schmitt, L. C., Ford, K. R., Rauh, M. J., Myer, G. D., Huang, B., & Hewett, T. E. (2010). Biomechanical measures during landing and postural stability predict second anterior cruciate ligament injury after anterior cruciate ligament reconstruction and return to sport. American Journal of Sports Medicine, 38(10), 1968–1978. 20702858 10.1177/0363546510376053 PMC4920967

[ref34] Spencer, M., Bishop, D., Dawson, B., & Goodman, C. (2005). Physiological and metabolic responses of repeated-sprint activities:specific to field-based team sports. Sports Medicine (Auckland, N.Z.), 35(12), 1025–1044. 16336007 10.2165/00007256-200535120-00003

[ref35] Verkhoshansky, Y. (2009). *Supertraining* (6th ed.). Verkhoshansky.

[ref36] Viera, A. J., & Garrett, J. M. (2005). Understanding interobserver agreement: The kappa statistic. Family Medicine, 37(5), 360–363. 15883903

